# High-Pressure Processing Effects on Microbiological Stability, Physicochemical Properties, and Volatile Profile of a Fruit Salad

**DOI:** 10.3390/foods13091304

**Published:** 2024-04-24

**Authors:** Ana C. Lopes, Rui P. Queirós, Rita S. Inácio, Carlos A. Pinto, Susana Casal, Ivonne Delgadillo, Jorge A. Saraiva

**Affiliations:** 1 Associated Laboratory for Green Chemistry-Network of Chemistry and Technology (LAQV-REQUIMTE), Department of Chemistry, University of Aveiro, 3810-193 Aveiro, Portugal; ana.celina@ua.pt (A.C.L.); carlospinto@ua.pt (C.A.P.); ivonne@ua.pt (I.D.); 2 Department of Applications and Food Processing, Hiperbaric S.A., Calle Condado de Treviño, 6, 09001 Burgos, Spain; r.queiros@hiperbaric.com; 3 School of Agriculture (ESA), Polytechnique Institute of Beja, Rua Pedro Soares, 7800-295 Beja, Portugal; rita.inacio@ipbeja.pt; 4 LAQV-REQUIMTE, Laboratório de Bromatologia e Hidrologia, Faculdade de Farmácia, Universidade do Porto, 4050-313 Porto, Portugal; sucasal@ff.up.pt

**Keywords:** high-pressure processing, fruit salad, total antioxidant capacity, polyphenol oxidase, microbiological stability, volatile profile

## Abstract

Nowadays, consumers are more aware of the effects of their diet on their health, and thus demand natural or minimally processed food products. Therefore, research has focused on processes that assure safe products without jeopardizing their nutritional properties. In this context, this work aimed to evaluate the effects of high-pressure processing (550 MPa/3 min/15 °C, HPP) on a fruit salad (composed of melon juice and pieces of Golden apple and Rocha pear) throughout 35 days of storage at 4 °C. For the physicochemical properties analysed (browning degree, polyphenol oxidase activity, antioxidant activity (ABTS assay), and volatile profile), a freshly made fruit salad was used, while for the microbiological tests (total aerobic mesophiles, and yeast and moulds) spoiled melon juice was added to the fruit salad to increase the microbial load and mimic a challenge test with a high initial microbial load. It was determined that processed samples were more microbiologically stable than raw samples, as HPP enabled a reduction of almost 4-log units of both total aerobic mesophiles and yeasts and moulds, as well as an almost 1.5-fold increase in titratable acidity of the unprocessed samples compared to HPP samples. Regarding browning degree, a significant increase (*p* < 0.05) was observed in processed versus unprocessed samples (roughly/maximum 68%), while the addition of ascorbic acid decreased the browning of the samples by 29%. For antioxidant activity, there were no significant differences between raw and processed samples during the 35 days of storage. An increase in the activity of polyphenol oxidase immediately after processing (about 150%) was confirmed, which was generally similar or higher during storage compared with the raw samples. Regarding the volatile profile of the product, it was seen that the compounds associated with melon represented the biggest relative percentage and processed samples revealed a decrease in the relative quantity of these compounds compared to unprocessed. Broadly speaking, HPP was shown to be efficient in maintaining the stability and overall quality of the product while assuring microbial safety (by inactivating purposely inoculated microorganisms), which allows for longer shelf life (7 versus 28 days for unprocessed and processed fruit salad, respectively).

## 1. Introduction

Fruits are an integral part of a healthy diet since they are a source of vitamins, minerals, antioxidants, phytochemicals, sugars, and dietary fibre, among others [1]. As per the World Health Organization, inadequate intake of fruits and vegetables contributes to around 1.7 million deaths, accounting for approximately 2.8% of total fatalities and ranking among the top 10 selected risk factors for global mortality. Melon (*Cucumis melo* L.) is a highly perishable fruit given its low acidity (pH > 4.6), its high water activity, and its matrix, which provides a good environment for bacterial growth, especially during cutting prior to consumption or if the surface of the melon suffers damage [2]. Apples (*Malus domestica*) are the second most consumed fruits in the USA and are considered the fourth most important fruits worldwide [3]. Pear (*Pyrus communis* L.) is very popular due to its desirable taste and high digestibility and its production represents a significant economic activity to Portugal (around 190,000 tonnes per year), where the exclusive Portuguese cultivar Rocha accounts for 95% of the national production [4].

The increased awareness of consumers regarding diet and health has led to a greater exploration of alternative food processing technologies. These must ensure the products’ microbial safety whilst preserving both the sensory and nutritional characteristics, allowing consumers to obtain products more similar to fresh foods [5,6]. High-pressure processing (HPP) is a nonthermal alternative for the extension of the shelf life of fruit-based products that are gaining popularity in the food industry and is considered one of the most important innovations in food processing during the past 50 years [7]. HPP uses a pressure-transmitting medium, usually water, to instantaneously transmit isostatic pressure (up to 600 MPa at industrial level) to food, at cold, room, or mild temperatures (about 60 °C), independently of size, shape, or composition of the food product and is usually employed in batch equipment [1]. HPP treatments are effective in inactivating most pathogenic and spoilage vegetative microorganisms and may considerably reduce the enzymatic activity in acid fruit juices and fresh fruits, without greatly affecting vitamins, pigments, volatile organic compounds, flavour, and nutritional value [8]. HPP is nowadays industrially applied in a wide range of products such as fruit juices, sea foods, meat, fruit–vegetable products, ready-to-eat foods, salads, sauces, and even pet foods [8], which are also promising when it comes to the impact on the environment and energy costs [9].

Polyphenol oxidase (PPO) (EC 1.14.18.1) reacts with phenolic compounds in the presence of oxygen and shows optimum activity at pH values between 5 and 7 [5]. It is found in fruits and vegetables and causes enzymatic browning when these foods are bruised or chopped, as well as browning discolouration during processing and storage. The colour of natural fruit products can primarily undergo changes as a result of the interaction between polyphenol compounds and PPO. Furthermore, it is widely assumed that PPO also plays a role in the oxidative breakdown of ascorbic acid [5]. Concerning the HPP effect on this enzyme’s activity, the results reported in the literature are not consistent [10,11,12,13].

Broadly speaking, PPO is very difficult to inactivate using HPP [14]. In fact, in studies regarding cloudy apple juice [15,16], it is possible to infer that when using moderate/high temperatures (>50 °C) in combination with pressures above 450 MPa, there is a higher efficiency in inactivating PPO. However, the sensorial aspects of the product are highly affected when using high temperatures, which needs to be taken into consideration. Despite the difficulty involved in comparing different products and different pressurizing conditions, the literature shows that PPO remains active in fruit treated by HPP, even if it exists in small percentages [17]. 

The sensory perception and consumer acceptability of foods are greatly influenced by their colour and volatile profile, which play crucial roles in the organoleptic quality of fruits and their derived products. HPP do not possess inhibitory effects on browning, as previously stated, but they do exhibit a lesser degree of browning compared to thermal processing due to the absence of Maillard reactions that occur as a consequence of thermal processing [18,19].

The volatile profiles of fruit are complex and depend on the cultivar, ripeness, pre- and post-harvest conditions, the fruit sample itself, and analytical methods utilized [20]. Volatile organic compounds (VOCs) produced in fresh fruits are mainly formed from fatty acids or amino acids and comprise various classes of chemicals, including esters, alcohols, aldehydes, ketones, lactones, and terpenoids [20,21]. Even though the number of chemical compounds identified as volatile organic compounds in fresh fruit is vast, only a fraction of these compounds are considered to have an impact on fruit flavour, based on their quantitative abundance and thresholds. Esters, for instance, are important volatile organic compounds in many fruits, conferring a distinct “fruity” odour [21]. 

Diverse VOCs commonly identified in fruit, namely in the fruits used in the present study to prepare fruit salads (apple, pear, and melon), were selected from the available literature and are displayed in Table S1 (Supplementary Materials).

This study aims to assess the effects of HPP (550 MPa, 3 min) on mixed fruit salads composed of apples, pears, and melon, focusing on changes in physicochemical properties, enzymatic activity, and microbial stability. This study also investigates the potential benefits of adding ascorbic acid (AA) as an antioxidant. The objectives include evaluating HPP’s impact on quality and shelf life, the enzymatic activity of polyphenol oxidase, the effect of AA addition, changes in volatile organic compounds, and the total antioxidant capacity. For the microbiological tests, spoiled melon juice was added to the fruit salad to increase the initial microbial load to evaluate the extension of the inactivation effect on microorganisms yielded by HPP.

## 2. Materials and Methods

Our study employed a controlled, comparative design to assess the impact of HPP (550 MPa, 3 min) on the microbial stability, physicochemical properties, enzymatic activity (focusing on polyphenol oxidase), and volatile organic profiles in a mixed fruit salad comprising Golden Delicious apples, Rocha pear, and melon juice. This study was designed to compare the effects of HPP treatment against a control group (unprocessed samples) stored under identical conditions.

### 2.1. Reagents and Solutions

2,2′-azinobis(3-ethylbenzthiazolin-6-sulfonate) (ABTS), 6-hydroxy-2,5,7,8-tetramethylchroman-2-carboxylic acid (TROLOX), 4-methylcatechol, and 2-phenylethanol were obtained from Sigma-Aldrich (Seelze, Germany). Potassium persulphate and absolute ethanol were purchased from Carlo ERBA Reagents (Val de Reuil, France). Sodium hydroxide was purchased from VWR (Leuven, Belgium). Sodium phosphate was purchased from Acros Organics (Geel, Belgium). Sodium dihydrogen phosphate anhydrous was purchased from Scharlau (Barcelona, Spain). Plate count agar (PCA) and rose bengal chloramphenicol agar (RBCA) were acquired from Liofilchem (Teramo, Italy), while Ringer tablets were purchased from Merck (Darmstadt, Germany). Food-grade ascorbic acid (AA) was kindly supplied by Nutre (Vagos, Portugal).

### 2.2. Fruit Salad Preparation

Golden delicious apples (*Malus domestica*), Rocha pear (*Pyrus communis* L.), and melons (*Cucumis melo* L.) grown in Portuguese territory were purchased at commercial maturity from a local supermarket and kept at 4 °C until use. 

**Figure 1:**
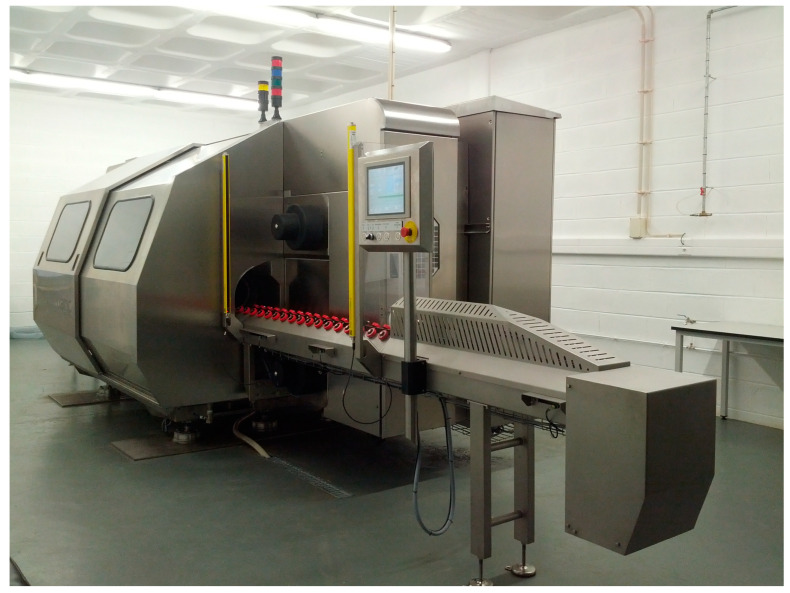
High-pressure processing (HPP) equipment where the fruit salad samples were processed at the Chemistry Department at the University of Aveiro, Portugal.

The fruits were washed in running water and manually peeled and ginned (to take out the lumps/pits of the fruit). Apples and pears were cut into uniform pieces (cylindrical pieces measuring 1 cm diameter and 0.5 cm thickness) and the pieces of melon were crushed with a blender (Braun MR 6500/500, Braun GmbH, Kronberg, Germany) to produce a juice. Then, the samples were prepared by mixing ca. 40 mL of melon juice with 4 pieces of apple and 4 pieces of pear in 60 ml flasks (Thermo Scientific™ Nalgene™ Wide-Mouth Lab Quality HDPE Bottles, Thermo Fisher Scientific Inc., Waltham, MA, USA) (destined for preparation for physicochemical and enzymatic analysis), or by mixing ca. 20 mL of melon juice with 2 pieces of apple and 2 pieces of pear in 30 mL flasks (destined for microbiological testing). The control group was immediately stored at 4 °C, and the HPP samples were immediately processed at 550 MPa for 3 min, at 15 °C using a pilot-scale HPP unit with a pressure vessel with 55 L of capacity (Model 55, Hiperbaric S.A., Burgos, Spain), which is shown in Figure 1, and afterward stored at 4 °C. This HPP equipment has a pressure vessel with an inner diameter of 200, a length of 2000 mm, and a maximum operation pressure of 600 MPa. The HPP equipment was connected to a refrigeration unit (RMA KH 40 LT, Ferroli, San Bonifacio, Italy) that allows for control of the temperature of the input water used as a pressurizing fluid.

In order to simulate a challenge test on the extension of the microbiological inactivation by HPP, and only for the samples used for the microbiological analyses, spoiled melon juice was added to the fruit salad in order to achieve the initial load of 5.50 log of total aerobic mesophiles (TAMs) and 2.17 log of yeasts and moulds (YMs).

A second assay was performed with the addition of AA to the samples prepared the same way as the ones previously described but food-grade AA was added in the concentration of 100 mg/kg [22] to the melon juice. Then, ca. 20 mL of melon juice with AA was mixed with 2 pieces of apple and 2 pieces of pear in 30 mL flasks. The control group was immediately stored at 4 °C, and the HPP samples were immediately processed at 550 MPa for 3 min, at 15 °C, and stored at 4 °C. In summary, two main sets of experiments were prepared, namely (1) control group—samples stored immediately at 4 °C without HPP treatment; and (2) HPP-treated group—samples subjected to HPP at 550 MPa for 3 min at 15 °C, and then stored at 4 °C. Additionally, a second assay involved adding ascorbic acid (AA) to the melon juice to assess its effect on the HPP-treated samples. The analyses were performed on duplicated samples from a single HPP cycle. This approach could be a weakness considering the low number of independent replicates; however, considering that this study aimed to only provide first insights on the potential use of HPP in a fruit salad, as the reader will find, it is reasonable to conclude that the preliminary findings suggest promising outcomes. Further studies with larger sample sizes and multiple HPP cycles are recommended to validate these initial observations and to explore the broader implications of HPP technology in enhancing the quality and shelf life of fruit salads.

### 2.3. Sample Clarification

To clarify the samples for physicochemical and enzymatic analysis, the samples in 60 mL bottles were first ground using a manual grinder and then homogenized (Miccra D-9 Homogenizer, Miccra GmbH, Heitersheim, Germany). Afterwards, the samples were centrifuged at 11,600 rpm, 4 °C, for 20 min (Heraeus Biofuge Stratos Centrifuge, Thermo Electron Corporation, Osterode, Germany). The supernatant was filtered (MN 640 w) and stored at −80 °C until further use.

### 2.4. Browning Degree

The browning degree value was determined by measurement of the absorbance of the samples at 420 nm in a UV–VIS spectrophotometer (Microplate Spectrophotometer Multiskan Go, ThermoScientific, Waltham, MA, USA) [23]. Higher values of absorbance at 420 nm correspond to higher browning.

### 2.5. Titratable Acidity and pH

The pH value of the samples was measured at 25 °C with a properly calibrated glass electrode (pH electrode 50 14, Crison Instruments, S.A., Barcelona, Spain). Titratable acidity (TA) was determined by titrating 25 mL of diluted sample (1:10) to pH = 8.9 with a standardized 0.01 M sodium hydroxide solution, using an automatic titrator (Titromatic 1S, Crison Instruments, S.A., Barcelona, Spain) based on AOAC Official Method 942.15 [24]. The results were expressed as g citric acid/g of fruit salad. The following equations were used to calculate the results:

(1) $$TA\;\left(M\;citric\;acid\right)=\left(\frac{\left[NaOH\right]\times V_{NaOH}(ml)}{V_{sample}(ml)}\right)÷\frac{dilution\;factor}{3}$$

(2) $$TA\;\left(g\;citric\;acid/L\right)=TA\;\left(M\;citric\;acid\right)\times {MW}_{citric\;acid}$$

To convert to g citric acid/g fruit salad, the relationship between the weight and extract volume of each sample was used.

### 2.6. Total Soluble Solids 

Total soluble solids (TSS) content was determined by measuring the Brix degree at 20 °C based on the AOAC Official Method 932.12 (AOAC International, 1932) [25] and the results were expressed as °Brix.

### 2.7. Total Antioxidant Capacity

Total antioxidant capacity (TAC) of the clarified samples was measured according to the method described by Kim et al. (2021) [26]. This method allows quantification of both water and lipid-soluble antioxidants via direct production of the ABTS^•+^ chromophore (blue/green) by reaction of ABTS and potassium persulfate. The ABTS^•+^ solution was prepared by addition, in a proportion of 1:1 (*v*/*v*), of 7 mM of ABTS diammonium salt to 2.45 mM of potassium persulfate, which was then left to react in the dark for 16 h. In order to obtain an absorbance of 0.700 ± 0.020, at 734 nm, the ABTS^•+^ solution was duly diluted in distilled water. An amount of 120 mL of the sample was added to 2 mL of a diluted ABTS^•+^ solution. After reacting in the dark for 6 min, the absorbance at 734 nm was measured using a UV–VIS spectrophotometer (Microplate Spectrophotometer Multiskan Go, ThermoScientific, Waltham, MA, USA). The analysis used a calibration curve with Trolox as a standard (ranging from 0 to 100 mg/mL). The outcomes were reported as Trolox equivalent antioxidant activity (TEAC) in mg/g of the fruit salad.

### 2.8. Polyphenol Oxidase

PPO activity was assayed based on the method described by Juarez-Enriquez et al. (2015) [27], but with slight modifications. First, 43 mL of sample was mixed with 130.0 mL of 50 mM sodium phosphate buffer (pH 6.5) and incubated at 25 °C. This mixture was considered the blank. Then, 87 mL of 4-methylcathecol 50 mM (substrate) was added and the absorbance was measured at 420 nm, 25 °C, every 10 s for 3 min using a UV–VIS spectrophotometer (Microplate Spectrophotometer Multiskan Go, ThermoScientific, Waltham, MA, USA). Enzymatic activity was expressed as DAbs/min. 

### 2.9. Microbiological Analysis

To carry out the analysis, each sample was aseptically homogenized with Ringer’s solution in a proportion of 1:10 in a Stomacher homogenizer (Stomacher 80 Biomaster; Seward Laboratory Systems Inc., Davie, FL, USA) for 3 min at high speed. Then, further decimal dilutions were carried out and droplets (20 µL) of the dilutions were plated on the surface of proper media in triplicate from duplicated samples (two fruit salad flasks) based on the colony count method of Miles and Misra, as reported by Inácio et al. (2022) [28]. 

Total aerobic mesophiles (TAMs) were enumerated in plate count agar (PCA) after incubation at 30 ± 1 °C for 72 ± 3 h (ISO 4833-2:2013) [29], and yeasts and moulds (YMs) were counted on rose bengal chloramphenicol agar (RBCA) after incubation at 25 ± 1 °C for 5 days (ISO 21527-1:2008) [30]. The results were expressed as logarithmic colony-forming units (CFU) per mL of blended fruit salad (log CFU/mL), with the plates being considered countable when the CFU ranged between 10 and 100. When no counts were found in the lowest serial dilution (corresponding to the direct plating of the juice on agar plates), the detection limit was 1.70 log CFU/mL according to the Miles and Misra method, while when the plates presented between 1 and 10 colonies in the lowest serial dilution, the quantification limit was 2.70 log CFU/mL. 

### 2.10. Volatile Organic Compound Analysis

Volatile organic compound analysis was performed by gas chromatography–mass spectroscopy (GC-MS) as described by Amaro et al. (2013) [31], with slight modifications, using a 7890A gas chromatograph coupled to a 5977 B mass selective detector, both from Agilent Technologies (Santa Clara, CA, USA). Control samples stored for 0, 3, and 7 days and HPP samples stored for 0, 3, 7, 14, and 21 days were analysed.

Fruit salads were homogenized using glass spheres and a vortex. A 2.5 g amount of pulp was weighted in 20 mL headspace precision thread Vials (LA-PHA-PACK, GMBH, Kronberg, Germany) and mixed with 25 μL of 2-phenylethanol (internal standard) prepared at 0.5 mg/mL in water, followed by 500 μL of NaCl 20% (*w*/*v*) to facilitate the volatile release to the headspace. The vials were sealed using magnetic screw caps with silicone transparent blue/PTFE white septa (LA-PHA-PACK) and placed in a heating plate at 40 °C for 40 min to equilibrate the headspace. The HS-SPME procedure was carried out using a 50/30 µm (1 cm) preconditioned divinylbenzene–carboxene–polydimethylsiloxane (DVB/CAR/PDMS) Stableflex 24 Ga fibre (Supelco, Bellefonte, PA, USA), which was in the injection port at 270 °C for 1 h, according to manufacture instructions. The SPME fibre was exposed to the headspace for 30 min, with absorbing volatiles at 40 °C. After extraction, the volatiles were desorbed from the SPME fibre into the gas chromatograph injection port set at 250 °C for 10 min, equipped with an SPME/direct (Supelco, St. Louis, MO, USA) liner, in the splitless mode with a constant pressure of 14.9 psi. Volatiles were separated on a 30 m × 0.25 mm i.d. × 0.25 μm thickness ultra-inert capillary column (HP-5MS, Agilent Technologies). The carrier gas was helium with a nominal initial flow rate of 1.9 mL min^−1^. The initial oven temperature was 35 °C, followed by a ramp of 3 °C min^−1^ up to 75 °C, and then at 20 °C min^−1^ to reach a final temperature of 250 °C, which was held for 5 min, with a total analysis time of 30 min. Mass spectra were obtained by electron ionization (EI) at 70 eV, in a full scan mode, with a spectrum range of ion mass captured between 40 and 450 m/z and an average of 3.5 scans s^−1^ (sample rate of 2). The mass spectra were evaluated using Enhanced ChemStation software (Version F.01.03.2357, Agilent Technologies). The peaks were identified using a mass spectrometer (5977 B mass selective detector, Agilent Technologies) coupled to the gas chromatograph by comparison of experimental spectra with those of the National Institute for Standards and Technology (NIST MS version 2.2) data bank. Only compounds with a match above 860 were considered. Out of these, the most important compounds were selected based on their presence and relevance in the literature, which was presented previously in the introduction section. Of the selected VOCs, only 5 showed a match below 900. The results were expressed in relative percentage of the total area counts in the full scan mode, excluding the area occupied by the internal standard and are presented in Section 3.6. The results in mg/kg of internal standard equivalents are presented in the Supplementary Materials.

### 2.11. Statistical Analysis

All analyses were performed in triplicate from duplicated samples, originated from a single HPP cycle, and expressed as mean ± standard deviation. The results were statistically analysed using one-way analysis of variance (ANOVA) followed by Tukey’s honest significant differences test at 5% significance. 

## 3. Results

### 3.1. Microbiological Analysis

**Figure 2:**
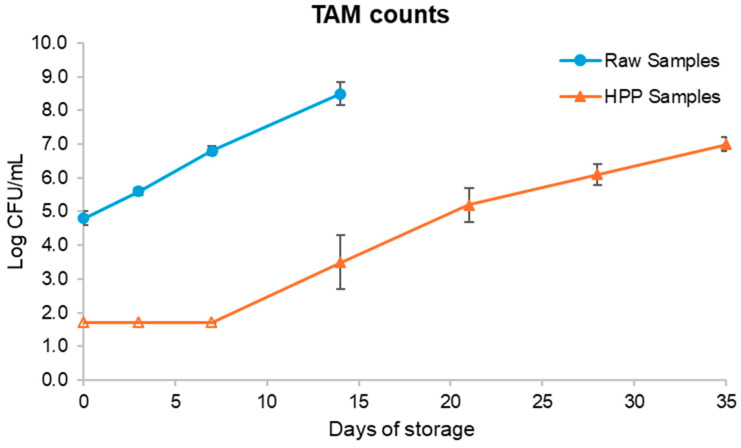
Graphic comparison of total aerobic mesophile counts over time for raw and high-pressure processing (HPP) samples. Raw samples stopped being analysed after day 14 given the extremely high microbial load. Empty-filled symbols mean that the detection limit (of 1.7 log CFU/mL) was reached.

**Figure 3:**
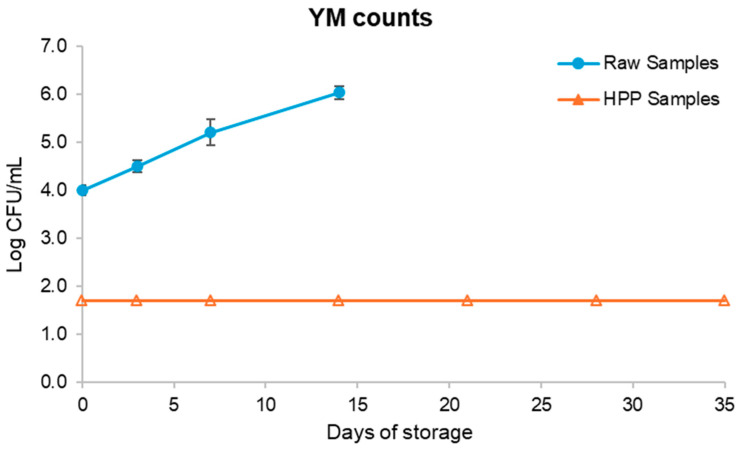
Graphic comparison of yeasts and mould counts over time for raw and high-pressure processing (HPP) samples. Raw samples stopped being analysed after day 14 given the extremely high microbial load. Empty-filled symbols mean that the detection limit (of 1.7 log CFU/mL) was reached.

TAM and YM growth during the shelf life evaluation period (35 days) were assessed as spoilage parameters. The results are presented in Figure 2 and Figure 3. The initial load of raw samples resulted from the inoculation previously mentioned. Before the inoculation with spoiled fruit salad, the initial TAM and YM counts were below the quantification limit (2.70 log CFU/mL), which aligns with previous reports from Martins et al. (2016) [32], who reported an initial psychrophile microbial load of 2.6 log CFU/mL in a fruit salad consisting of pineapple, banana, guava, apple, papaya, and mango. As such, the inoculation with spoiled fruit salad aimed to increase the microbial load to obtain a better understanding of the magnitude of the effect of HPP on the fruit salads’ microbial load reduction.

Therefore, it can be concluded that subsequent to the application of HPP treatments, there was a reduction in YM and TAM counts by approximately four logarithmic units, descending below the threshold of detection. Typically, the inactivation of microorganisms is achievable through the exertion of pressures within the range of 350 to 600 MPa [33]. HPP shows multi-targeted effects; for example, it induces the unfolding of globular proteins, induces the disintegration of ribosomes, affects metabolic pathways, and leads to an inability to control intracellular pH and proliferate among other essential processes. These effects are reversible at low pressures (<350 MPa) but irreversible at higher pressures, where, ultimately, the permeabilization of the cell membrane causes cell death [34]. 

During the pressurization stage, as the pressure level increases, the cellular functions become gradually compromised due to the inactivation of key enzymes, disruption of membranes, etc., leading to loss of cell viability. Hence, a pressure treatment of 550 MPa caused cell death [35]. Similar results were obtained in other fruit products; for instance, Chen et al. (2013) [36] compared the effects of 300 and 400 MPa in cloudy pomegranate juice under holding times between 2.5 and 25 min at room temperature. The results showed that the use of 400 MPa allowed shorter holding times and assured greater decimal cycle reductions. For example, for a holding time of 5 min, TAM counts showed a reduction of 4.53 log CFU/mL and YM counts decreased from 3.69 log CFU/mL to below the limit of detection. Using 300 MPa and the same holding time, these values were 3.23 log CFU/mL and 1.89 log CFU/mL, respectively. Varela-Santos et al. (2012) [37], in a study also examining pomegranate juice, reported that, in general, pressures from 350 MPa on are more effective in reducing the microbial loads to values below the limit of detection. This can be explained by the fact that the irreversible denaturation of proteins may occur above 300 MPa, which is one of the main reasons behind the inactivation of vegetative cells, as mentioned before [38].

Regarding YM content in HPP samples, the low value remained consistently stable over the course of 35 days of storage at a temperature of 4 °C. The absence of YMs can be attributed to their lesser tolerance to non-acidic conditions. Compared to the raw samples, YMs were already present in the inoculated samples, but their growth was less pronounced than TAMs. This could be attributed to the high concentration of bacteria, which may have hindered the growth of YMs. The non-acidic food matrix may have also exerted an influence [39]. 

At day 14, the raw samples were already highly contaminated, with 8.39 log CFU/mL regarding TAMs and 5.99 log CFU/mL regarding YMs, and showed clear signs of spoilage with an uncharacteristic and unpleasant odour. Given the high microbial load at this point, analysis of the raw samples was concluded at this stage. 

Concerning TAM counts on HPP samples, these only started being detected after 14 days of cold storage and remained within the acceptable limits until the 21st day of cold storage. The samples analysed after 28 days of storage showed unpleasant odours, similar to raw samples on the 14th day of storage, which demonstrates that the microbial growth in HPP samples was slower than in raw samples. These results are backed by the TA results, which were presented previously, and together give a basis to infer that the product is microbiologically stable for 21 days after HPP at most. Therefore, it can be interpreted that HPP can indeed considerably reduce the microbial load in fruit products, which allows shelf life extension. Similar results can be found in the literature for other fruit products; for instance, Hurtado et al. [17,38] reported that HPP-processed red fruit-based smoothies were microbiologically stable and retained their “fresh-like” properties for at least 14 days at 4 °C. Accordingly, Queirós et al. [40] reported that sweet cherry juice subjected to HPP showed TAM and YM values below the limit of detection throughout 28 days of storage at 4 °C. Landl et al. (2010) [41] also concluded that HPP-processed acidified apple purée reached 3 weeks of refrigerated storage without microbial growth. Mota et al. (2013) [36] verified that HPP-processed pomegranate juice still met the Chinese hygienic standard for fruit juices (≤100 CFU/mL TAM and ≤20 CFU/mL YM) after 90 days of storage at 4 °C. These results, along with the results of Patterson et al. (2012) [42] and many other works not hereby mentioned, allow us to infer that the efficiency of HPP has been demonstrated, providing assurance of the microbial safety of fruit-based products, not only immediately after processing but also for a long period of time. 

Due to the inoculation, it is not possible to infer exactly for how many days the raw sample would be microbiologically stable, but due to the product’s low acidity and high water activity, it would not have been more than a few days. The literature states that the shelf life of minimally processed fresh-cut fruits is ≤6 days, showing that even though the worst-case scenario was simulated in the present study with a heavily contaminated fruit salad, HPP was still able to inactivate at least 3.3 log units of TAMs to below detection limits, remaining as so for up to 7 days of storage (where, in a typical case of a fresh unprocessed salad, after 7 days it would be spoiled according to the literature) [43]. Considering that YM counts remained below detection limits during all the storage periods evaluated and that TAM counts grew above 5 log CFU/mL after 21 days, a considerable shelf life extension yielded by HPP of at least 15 days was possible if we consider the data available in the literature regarding the shelf life of a minimally processed fruit salad previously mentioned.

### 3.2. Total Soluble Solids, pH, and Titratable Acidity

The initial TSS was 10.80 ± 0.20 and 11.07 ± 0.12 °Brix for raw and HPP samples, respectively. The results show that TSS content did not suffer significant changes (*p* > 0.05), neither derived from the storage time nor derived from the subjection to HPP (these results are available in Table S2 of the Supplementary Materials). During storage, there were slight fluctuations among the processed samples, while the raw juice presented a slight decline. This was also verified by Queirós et al. (2015) [40] and Chen et al. (2013) [36], and both works concluded that HPP had no significant effect on TSS (*p* > 0.05). Wolbang et al. (2008) [44] studied the effect of HPP on the nutritional value and quality attributes of *Cucumis melo* L. and came to the same conclusion that TSS was not significantly affected by HPP (*p* > 0.05). 

**Figure 4:**
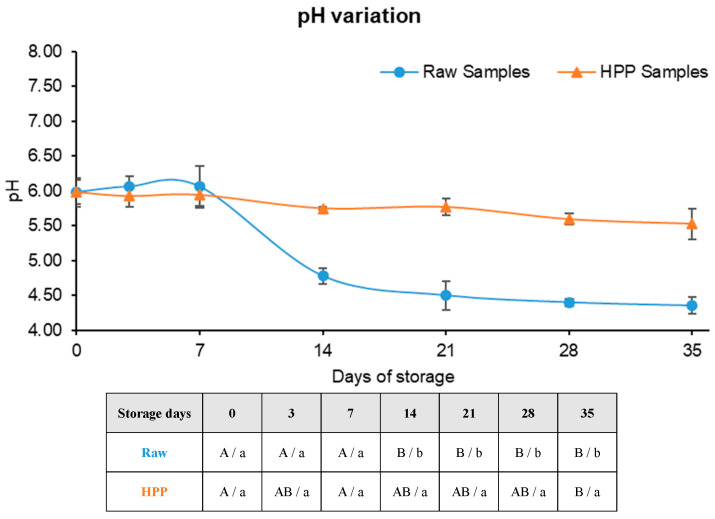
pH variation through time in cold storage and respective one-way ANOVA results table. Different letters represent significant differences (*p* < 0.05) at the same conditions (capital letters: effect of storage) or between samples at the same time of storage (noncapital letters: effect of high-pressure processing, HPP).

The pH of both raw and HPP samples was approximately 6 (Figure 4), confirming the non-acidic profile of this product. 

Initially, the pH levels of the unprocessed samples were elevated compared to those subjected to HPP, a variation that could be attributed to the processing method itself. It is posited that the application of high pressure may induce the dissociation of water molecules into ions, resulting in a reduction in the pH value [1].

**Figure 5:**
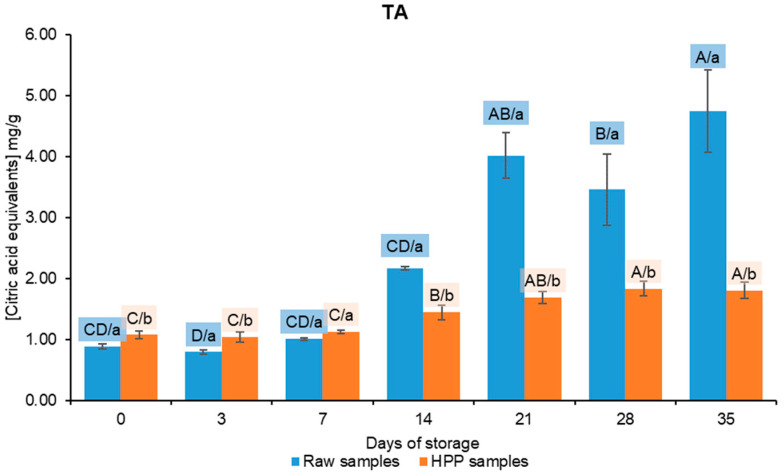
Titratable acidity results and respective one-way ANOVA results. Different letters represent significant differences (*p* < 0.05) at the same conditions (capital letters: effect of storage) or between samples at the same time of storage (noncapital letters: effect of high-pressure processing, HPP).

These samples suffered an accentuated decrease (*p* < 0.05) in pH between the 7th and 14th day stored at 4 °C, which is most likely associated with the increasing microbial activity during this period. Regarding HPP samples, the pH decreased slowly over time. These results are concordant with TA results, which are summarily presented in Figure 5. 

In fact, there is a large correlation (|R| = 0.938, *p* = 0.002) between pH and TA values, both in raw and HPP samples.

When comparing the total acidity (TA) in raw and high-pressure processing (HPP) samples, there is a statistically significant change (*p* < 0.05). However, the difference in TA is slight and likely due to a slow leakage of organic acids from the vegetable cell organelles into the juice matrix after HPP [45]. However, starting with the 14th day, it becomes easy to observe a significant difference (*p* < 0.05) between the two groups of samples, indicating a disparity in microbial quantity. Raw samples exhibit a more rapid increase in acidity compared to HPP samples, mostly because of their elevated microbial count. Concerning HPP samples, the temperature of the samples begins to increase gradually after the 14th day when stored in a refrigerator. This corresponds to the time when microbiological counts started to discover colonies of TAMs. The section following this document presents the results of the microbiology stability analysis.

### 3.3. Browning Degree

A significant disparity in visual perception between raw and HPP samples was observed in the assay conducted without the inclusion of any antioxidant compound. Exemplifying images can be found in Figures S1 and S2 (Supplementary Materials). Instrumental results are presented and discussed subsequently. This effect has been documented in other studies, such as the studies of Wolbang et al. (2008) [44] and Zhang et al. (2017) [46].

**Figure 6:**
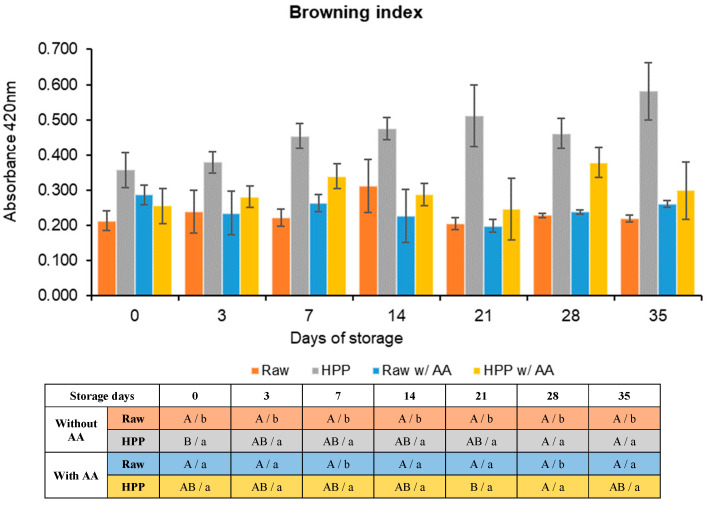
Browning index results regarding the assays with and without the addition of ascorbic acid (AA) and respective one-way ANOVA results. Different letters represent significant differences (*p* < 0.05) at the same conditions (capital letters: effect of storage) or between samples at the same time of storage (noncapital letters: effect of high-pressure processing, HPP).

When the ascorbic acid (AA) was added visually, there was still a perceptible difference in the colour, with the HPP samples being browner than the raw samples, although this difference was less intense than in the previous assay (juice images are provided in Figure S2 and instrumental results are shown in Figure 6). 

In the assay without AA, there was a significant difference (*p* < 0.05) between raw and HPP samples throughout the 35 days of storage. Raw samples did not show significant changes (*p* > 0.05) in their browning index during storage, while HPP samples showed a significant difference (*p* < 0.05) between day 0 (0.358 ± 0.050) and the 28th (0.461 ± 0.043) and 35th (0.581 ± 0.081) days of storage, showing a slow increase in browning index.

Regarding the analysis involving the inclusion of AA, the performance of untreated samples closely resembled the ones without AA considering the lack of changes (*p* > 0.05) in their browning degree throughout the storage period. Regarding HPP samples with AA, between those from day 0 to the 14th and 35th days, there are no significant changes. However, on the 28th day, there is a significant increase (*p* < 0.05) followed by a decrease at the 35th day. This sudden increase can be a consequence of the heterogeneity of the samples themselves.

Based on the graph presented above, it is possible to notice differences when comparing the two assays, namely between the bars representing HPP samples. For example, in a study performed by Guerrero-Beltrán et al. (2005) [47], it was observed that HPP-treated peach puree (517 MPa, 5 min, 25 °C) supplemented with AA preserved higher colour stability during 30 days of storage at 3 °C compared to unprocessed (but supplemented) peach puree. Yet no significant differences were found in colour stability for HPP samples regardless of being supplemented with AA or not, i.e., both supplemented and non-supplemented samples presented similar colour stability, which aligns with the findings of the present study.

### 3.4. Total Antioxidant Capacity

**Figure 7:**
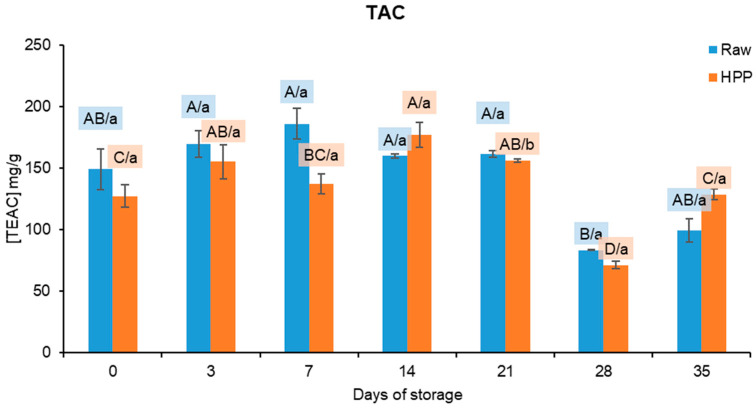
Antioxidant activity expressed as mg of Trolox equivalent antioxidant capacity (TEAC)/g. Different letters represent significant differences (*p* < 0.05) at the same conditions (capital letters: effect of storage) or between samples at the same time of storage (noncapital letters: effect of high-pressure processing, HPP).

The graphical representation of the TAC results can be observed in Figure 7. While the quantification of total phenolics (TPs) and vitamin C was not feasible, the detection of antioxidant activity in the samples was possible. The nutritional quality of melon is directly associated with its high quantity of b-carotene. Since melon juice is the main ingredient in the fruit salad being tested, it is possible that these chemicals are responsible for the TAC [48]. According to Rúa et al. (2018) [49], “Piel de sapo” melon juice has 0.107 ± 0.012 mg TEAC/g, which is in the same range of values determined in this work. 

Considering the results, these did not show a clear tendency. While there were minor fluctuations in the results over time, there were no statistically significant disparities (*p* > 0.05) between the raw and HPP samples, except for the samples analysed after 21 days of refrigerated storage. Statistical analysis revealed significant differences (*p* < 0.05) between the TEAC concentration of the raw and HPP samples, despite their apparent similarity in the graphical display of the results. 

The storage time did not have a significant influence (*p* > 0.05) on the TAC in raw samples up to the 28th day. The fact that only the samples from this day revealed significant changes may be a consequence of the samples’ heterogeneity. 

Regarding the data obtained from HPP samples, they do not evidence a distinct pattern, yet they display a similar overall evolution to that observed for raw samples. Furthermore, as previously noted, there are no significant differences between the HPP samples and raw samples overall. Thus, it may be deduced that HPP does not exert a significant impact on the antioxidant activity of the samples, as shown in previous research. For instance, Mukhopadhyay et al. (2017) [50] evaluated the effects of HPP (300–500 MPa for 5 min) and subsequent storage at 4 °C for 10 days on the antioxidant activity of cantaloupe melon puree. The authors reported no significant differences between unprocessed and processed samples and during storage, regardless of the processing conditions. Yet the effects of HPP on fruit juices and pulps are quite dependent on the fruit mixture, processing conditions, subsequent storage conditions, etc. [16].

### 3.5. Polyphenol Oxidase Activity

**Figure 8:**
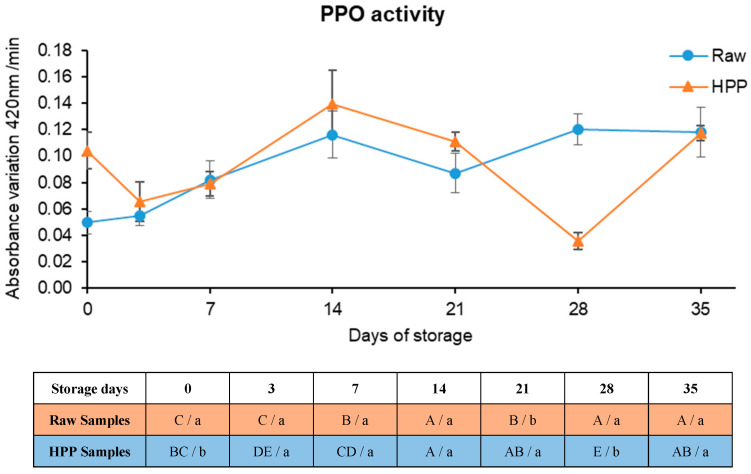
Polyphenol oxidase activity variation through time in cold storage and respective one-way ANOVA results table. Different letters represent significant differences (*p* < 0.05) at the same conditions (capital letters: effect of storage) or between samples at the same time of storage (noncapital letters: effect of high-pressure processing, HPP).

The HPP samples exhibited a higher level of PPO activity immediately after processing (0.1041 ± 0.0084 Abs/min) compared to the raw samples (0.0496 ± 0.0136 Abs/min), as seen in Figure 8, which correlates to the lower browning index observed for unprocessed samples (as seen in Figure 6). When under pressure, even though covalent bonds are not changed, the key stabilizers of the three-dimensional conformation of the enzyme, such as disulphide bonds, hydrogen bonds, and hydrophobic, electrostatic, and van der Waals interactions, are disturbed. These modifications could lead to a rise or fall in biological activity and could modify the substrate specificity [5]. Pressure has been reported to stimulate the activity of certain enzymes, particularly monomeric enzymes such as PPO [5]. Moreover, HPP destabilizes the compartmentalization in the intact cells of the substrates and enzymes, leading to their interaction [51]. These results are in accordance with other reports where HPP was performed near room temperature [27]. In order to achieve higher PPO inactivation, higher temperatures should be used [52]; however, this would go against the purpose of maintaining the fresh-like attributes of the product and would also make the process less economically attractive and environmentally friendly. 

By the 3rd day of storage experiments, the residual activity of PPO decreased abruptly in HPP samples while raw samples showed a slight increase, resulting in similar activity (*p* > 0.05) in both samples. From then on, PPO activity showed no significant differences (*p* > 0.05) between raw and HPP samples, except for the samples taken on the 21st and 28th days. This may be caused by the heterogeneity of the samples themselves. 

Falguera et al. (2013) [53] studied PPO inactivation in apple juices made from six apple varieties. Looking at the results of their study, it is possible to conclude that, in general, apple PPO is extremely pressure-resistant if the process is carried out at room-like temperatures (25 °C) since the maximum inactivation achieved (after 16 min at 600 MPa) in Golden Delicious PPO was only 7% (presenting a residual activity 93%), as such, being one of the most resistant. Hurtado et al. (2015) [11] observed no effect of HPP on PPO activity in fruit smoothies, unlike [12], where higher inactivation was achieved at near-ambient temperatures. However, the authors did not present any explanation for this disparity in results. Rao et al. (2014) [10] reported 79% inactivation of PPO in peach juice at 600 MPa/25 min/25 °C, even though the residual activity increased ca. 7.3% after processing at 400 MPa for 5 min. This activation of PPO has been observed in other products, such as cloudy apple juice [15]. The most pertinent explanation for this phenomenon, which has been verified, is that there are two PPO isoforms: one isoenzyme is sensitive to pressure and the other is stable [10,15]. 

Considering the aforementioned results, it is possible to state that PPO did not suffer HPP inactivation and was the major contributor to the extreme browning observed.

### 3.6. Volatile Organic Compounds

**Figure 9:**
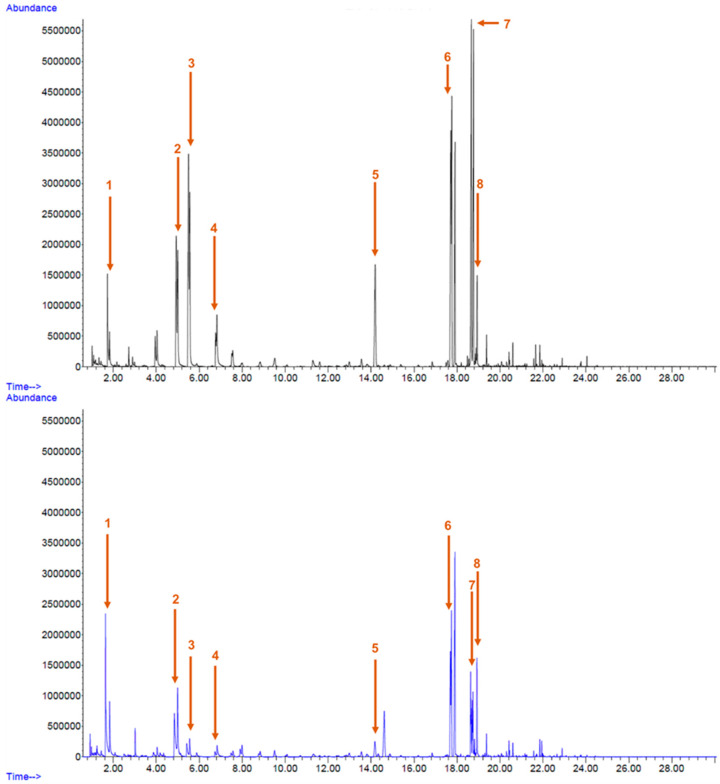
Total ion chromatograms referring to samples from day 0. The black line refers to a raw sample, while the blue line refers to an HPP sample. 1—ethyl acetate; 2—hexanal; 3—butyl acetate; 4—(E)-2-hexenal; 5—hexyl acetate; 6—(Z)-6-nonenal; 7—(E,Z)-2,6-nonandienal; and 8—(E)-Z-nonenal.

The main VOCs identified in fruit salads composed of melon juice and pieces of apple and pear, without the addition of AA, are presented in Table 1. Also, two chromatograms, in which the peaks representing bigger areas with clear differences between the two groups of samples were highlighted, are presented in Figure 9 as an example (these chromatograms refer to samples of day 0). 

Considering that apple and pear were not the main constituents of the fruit salad and were present as solid pieces while the melon was in the form of a liquid, it was not anticipated that the characteristic VOCs of apple and pear would significantly contribute to the olfactory profile of the product. Volatile chemicals are a significant focus in this study, given that melon juice is the primary constituent of the fruit salads under investigation.

The majority of the volatile organic compounds (VOCs) detected in both unprocessed and HPP samples were aldehydes, followed by acetate esters. Additional classes were discovered, although their representation was considerably less. 

Regarding aldehydes in raw samples, these showed a gradual relative reduction during storage at 4 °C, as observed in melons in the work of [54]. The relative percentage was significantly (*p* < 0.05) higher on day 0 when compared with the 7th day. Concerning the impact of HPP, samples subjected to HPP presented a significantly (*p* < 0.05) lower relative percentage of aldehydes immediately after processing than raw samples (43.3 ± 2.8 versus 55.4 ± 2.9%, respectively). The relative amounts in HPP samples showed a slow tendency to increase, given that on the 14th day of storage it had increased to 50.6 ± 3.2%. 

Acetate esters stood as the second most representative class of VOCs present in these fruit salads’ volatile profiles. This class showed a tendency to decrease over storage time in raw samples, being significantly lower (*p* < 0.05) after 7 days of refrigerated storage. In samples subjected to HPP, the proportions significantly increased (*p* < 0.05) immediately after processing in comparison with raw samples from day 0. Nevertheless, HPP samples showed the same tendency to decrease with storage. 

Oh et al. (2011) [55] also used HS-SPME in *Cucumis melo* L. and considered (Z)-6-nonenal, nonanal, (E,Z)-2,6-nonadienal, and (E)-2-nonenal characteristic impact flavour and aroma compounds (CIFAC) of melon. These compounds were also found in the samples analysed in this work in significant relative percentages. (Z)-6-nonenal did not show a clear tendency to increase or decrease with storage time in raw samples. However, there was a significant (*p* < 0.05) reduction (4% less) in HPP samples immediately after processing, and there was a clear decrease in the proportion of (Z)-6-nonenal over storage time. The proportion of nonanal did not show significant differences (*p* > 0.05) between raw and HPP samples, and in both groups of samples, a decreasing tendency was observed in nonanal with storage time. Regarding (E,Z)-2,6-nonadienal, there were no significant (*p* > 0.05) changes in raw samples over 7 days of storage at 4 °C. Nonetheless, a significant reduction (*p* < 0.05) was verified after HPP, having a 6% difference from raw samples in day 0. 

The content of (E,Z)-2,6-nonadienal in HPP samples increased during the 21 days of storage. Finally, (E)-2-nonenal showed a behaviour similar to (E,Z)-2,6-nonadienal, having undergone a significant reduction after HPP and increased storage time in processed samples. 

The alcohols correspondent to the previously highlighted aldehydes, namely 1-nonanol, (E)-2-nonen-1-ol, (Z)-3-nonen-1-ol, and (E, Z)-2,6-nonadien-1-ol, were detected in the studied fruit salads in much smaller proportions. Besides its association with melon flavour, 1-nonanol, (Z)-3-nonen-l-ol, and (E)-2-nonen-1-ol are also associated with pear flavour [56,57]. Different behaviours in raw and HPP samples were observed. The compound 1-nonanol was detected in both groups of samples; however, it increased over storage time in raw samples and decreased over time in HPP samples. (E)-2-nonen-1-ol was present in raw samples, and significantly increased but was not detected in samples subjected to HPP. The absence of this compound in samples subjected to HPP was also verified in other food matrices [58]. The content in (Z)-3-nonen-1-ol increased in raw samples, given it was not detected in the first 3 days of storage, but on the 7th day represented 0.48 ± 0.02%. In HPP samples, it was only detected in the first 3 days of refrigerated storage and in very small percentages (0.05 ± 0.00% and 0.06 ± 0.01%, respectively). The increase in the contents of these alcohols in raw samples may result from the reduction in the corresponding aldehydes [55]. However, these aldehydes are enzymatically reduced to their corresponding alcohols. Additionally, high pressure affects the activity of some enzymes, causing both activation and inactivation, depending on the enzyme itself, its origin, and its matrix, among others. The observed difference regarding the behaviour of the impact factors of melon characteristics and aroma compounds (CIFACs) and their corresponding alcohols in HPP samples may result in changes in the activity of enzymes involved in the biosynthetic pathways that originate these VOCs. For example, the quantity of (E)-2-nonenal increased over storage time while no (E)-2-nonen-1-ol was detected. This indicates that the metabolic pathway was somehow affected. 

Considering C6 aldehydes, hexanal, which is characteristic of the three fruits composing the fruit salad, decreased abruptly from day 0 to day 3 in raw samples and remained similar on the 7th day of storage. When comparing hexanal in raw and HPP samples on day 0, processed samples evidenced significantly (*p* < 0.05) higher proportions, as was verified by Navarro et al. (2002) [59] in strawberry purées and did not suffer significant changes (*p* > 0.05) during storage, and always remained significantly (*p* < 0.05) higher than raw samples. The fact that hexanal increases in HPP fruit salad may be related to cell disruption that leads to increased contact between enzymes and the substrates, as HPP cannot fully inactivate lipoxygenase (LOX), as described by Rodrigo et al. (2007) [60], who reported a residual activity of 20% for LOX in tomato juice after HPP (650 MPa, 12 min). On the other hand, in the present study, (E)-2-hexenal (that decreased during storage in raw samples) suffered a reduction immediately after HPP, contrary to that observed by Navarro et al. (2002) [59]. Nevertheless, our results align with those reported by Kebede et al. (2018) [61], which also reported a decrease in the relative peak area of (E)-2-hexenal right after HPP (600 MPa, 3 min) and a slight reduction during 5 weeks of refrigerated storage. Hexanal is a common volatile organic compound found in many fruits and vegetables and is known for its grassy or green odour. It is often formed as a breakdown product of linoleic acid, a polyunsaturated fatty acid, through enzymatic pathways involving LOX activity [62]. 

Regarding acetate esters, ethyl acetate and butyl acetate showed a significant increase in samples subjected to HPP. The content of ethyl acetate showed a twofold increase (approximately) on days 0 and 3 than in raw samples but did not suffer significant changes (*p* > 0.05) during storage in both groups of samples. Butyl acetate is usually found in great amounts in melon [54], and its content was also significantly (*p* < 0.05) greater in HPP samples and decreased over storage time in both groups of samples. Pentyl acetate and heptyl acetate both showed an increase during storage in raw samples but showed different behaviours in processed samples. Pentyl acetate registered a slow decrease through storage while heptyl acetate was only detected immediately after processing. The increasing relative percentage of these compounds in raw samples may be related to microbial activity, given the load these samples presented. If pentyl and heptyl acetate do result from microbial metabolism, the reduction in the microbial load in processed samples can also explain why their relative percentage did not increase. Moreover, according to Yi et al. (2018) [63], acetate esters’ decrease during storage may be linked to esterase activity. In fact, and as mentioned previously, high pressure can either activate or inhibit enzymatic activity. In this way, given the decrease in the quantity of these two acetate esters in HPP samples, the activation of esterase presents itself as a possible justification. 

Regarding the presence of furan-related compounds, these have been detected before in various thermally treated food products, and their formation is related to the Maillard reaction and oxidation of triple-unsaturated FA [64]. It was hypothesized that these could be artefacts formed by chemical reactions in the course of isolation of volatiles. However, given that these increased with time, this hypothesis was discarded. It was also hypothesized that these compounds could be a result of HPP. This theory was also discarded given the low temperature (15 °C) at which HPP was performed and given the presence of these compounds in raw samples. The only explanation left is that these compounds are naturally present in the samples [58]. 

Compounds typically associated with apple’s aroma, such as 2-hexen-1-ol acetate, hexyl 2-methylbutyrate, and α-pinene were detected in both raw and HPP samples, but in small percentages, as shown in Table 1. 2-hexen-1-ol acetate showed a slight increase throughout storage time in raw samples, but in HPP samples it decreased until it was no longer detected. Hexyl 2-methylbutyrate showed a significant (*p* < 0.05) increase in raw samples in the 7 days of storage at 4 °C. No significant difference (*p* > 0.05) was verified between the content in raw and HPP samples, having shown the same increasing tendency in processed samples up until the 7th day of storage, after which it abruptly decreased. α-pinene also significantly increased (*p* < 0.05) in raw samples after 7 days at 4 °C. The content of this terpene was significantly higher (*p* < 0.05) in HPP samples immediately after processing when compared with the content on day 0 in raw samples, and also increased with storage time. α-farnesene, present in apples and pears, was also found in small relative quantities, in both groups of samples. Similar to α-pinene, it increased with storage time in both groups of samples, but HPP did not have a significant (*p* > 0.05) impact on α-farnesene content immediately after processing. HPP did not show benefits regarding melon’s CIFAC, decreasing its abundance. Given that the HPP effect on the VOC profile of fruit products is very scarcely reported in the literature, further studies on this matter should be performed.

## 4. Conclusions

In this study, HPP was demonstrated to be efficient in preserving the overall quality of fruit salad (melon juice and pieces of apple and pear). Regarding total soluble solids and total antioxidant activity, HPP did not implicate major differences between the two groups of samples. Furthermore, HPP drastically reduced the microbial load in samples for up to 21 days. The differences in microbial activity may justify the differences in TA and pH. However, HPP was not efficient in inactivating PPO and results point to its activation since the browning index in processed samples was significantly higher (*p* < 0.05). The addition of an antioxidant, such as ascorbic acid, can help limit enzymatic browning and lead to benefits.

Regarding VOCs, it was verified that HPP samples had less content in melon characteristic impact flavour and aroma compounds than raw samples and different behaviours of these compounds and others related to them were observed. 

Considering that this study aimed to only provide first insights on the potential use of HPP in a fruit salad, it is reasonable to conclude that the preliminary findings suggest promising outcomes. Further studies with larger sample sizes and multiple HPP cycles are recommended to validate these initial observations and explore the broader implications of HPP technology in enhancing the quality and shelf life of fruit salads. Further experiments regarding the effect of HPP on TP content, vitamin C, and pectin methylesterase and peroxidase activities are needed in order to reach a better understanding of how fruit salads respond to this kind of processing. Future work should also comprise the quantification of b-carotene and the evaluation of alterations in the texture and colour of the solid components of the product, as well as sensorial analysis. Controlling the browning of the product is also mandatory in order to preserve its visual properties. Therefore, more concentrations of ascorbic acid must be tested. 

## Figures and Tables

**Table 1:** Main volatile organic compounds extracted by HS-PME measured by GC-MS. Results expressed in relative percentage of the total area counts in the full scan mode.

					Compound Relative Percentage (%)							
					Raw Samples Stored at 4 °C			HPP Samples Stored at 4 °C				
Compound Family	RT ^a^	Compound Name	CAS N°	RI ^b^	Day 0	3rd Day	7th Day	Day 0	3rd Day	7th Day	14th Day	21st Day
**Acetate esters**	1.72–1.88	Ethyl acetate	141-78-6	612	3.47 ± 0.66 A/b	2.74 ± 1.48 A/b	3.29 ± 0.28 A/a	8.04 ± 1.67 A/a	7.35 ± 0.99 A/a	4.80 ± 1.56 A/a	5.17 ± 0.50 A	5.13 ± 3.9 A
	2.88/2.98	Propyl acetate	109-60-4	708	0.42 ± 0.06 A	0.54 ± 0.01 A/a	0.52 ± 0.11 A/b	nd	0.56 ± 0.40 A/a	0.79 ± 0.04 A/a	0.30 ± 0.11 A	nd
	5.4-5.6	Butyl acetate	123-86-4	812	15.7 ± 0.44 A/b	14.2 ± 2.2 A/b	8.35 ± 1.75 B/b	19.7 ± 0.6 AB/a	21.5 ± 0.1 A/a	21.5 ± 0.1 A/a	13.4 ± 3.3 BC	11.2 ± 2.4 C
	7.9–8.05	2-methyl-1-butanol acetate	624-41-9	880	0.24 ± 0.01 A/b	0.13 ± 0.02 B/a	0.11 ± 0.06 B/a	1.76 ± 0.45 A/a	0.18 ± 0.05 B/a	0.15 ± 0.07 B/a	0.02 ± 0.01 B	nd
	9.4–9.6	Pentyl acetate	628-63-7	911	0.68 ± 0.05 B/a	1.20 ± 0.13 AB/a	1.77 ± 0.42 A/a	0.69 ± 0.22 A/a	0.46 ± 0.14 AB/b	0.49 ± 0.10 AB/b	0.26 ± 0.06 B	0.23 ± 0.07 B
	14.2	Hexyl acetate	142-92-7	1011	8.41 ± 2.32 A/a	6.85 ± 0.86 A/a	6.37 ± 1.12 A/a	5.02 ± 2.53 A/a	3.85 ± 0.78 AB/b	3.85 ± 0.78 AB/a	1.11 ± 0.11 B	0.64 ± 0.15 AB
	14.32–14.35	2-hexen-1-ol acetate	2497-18-9	1016	0.17 ± 0.09 B/a	0.39 ± 0.07 A/a	0.31 ± 0.03 AB/a	0.27 ± 0.04 A/a	0.10 ± 0.05 B/b	0.10 ± 0.05 B/b	nd	nd
	17.96	Heptyl acetate	112-06-1	1113	0.15 ± 0.01 C/b	0.49 ± 0.03 B	0.83 ± 0.11 A	0.21 ± 0.01 a	nd	nd	nd	nd
	20.42	6-nonenyl acetate	35854-86-5	1308	0.24 ± 0.03 A/b	0.23 ± 0.04 A/a	0.22 ± 0.02 A/a	0.62 ± 0.04 A/a	0.26 ± 0.03 B/a	0.26 ± 0.02 B/a	0.16 ± 0.02 C	0.10 ± 0.01 C
	20.44	Nonyl acetate	143-13-5	1308	0.10 ± 0.01 A	0.12 ± 0.02 A	0.14 ± 0.02 A	nd	nd	nd	nd	0.03 ± 0.00
	S acetate esters				29.3 ± 1.5 A/b	26.52 ± 3.2 AB/b	21.71 ± 3.5 B/b	36.45 ± 2.4 A/a	34.3 ± 0.7 A/a	31.8 ± 1.4 A/a	20.0 ± 2.8 B	17.3 ± 5.4 B
**Non-acetate esters**	19.73	Hexyl 2-methylbutyrate	10032-15-2	1236	0.02 ± 0.01 B/a	0.03 ± 0.01 AB/a	0.05 ± 0.02 A/a	0.03 ± 0.02 B/a	0.06 ± 0.03 AB/a	0.13 ± 0.05 A/a	0.01 ± 0.00 B	0.03 ± 0.01 B
**Aldehydes**	4.6–5.08	Hexanal	66-25-1	800	9.46 ± 0.26 A/b	2.28 ± 0.47 B/b	2.79 ± 0.69 B/b	12.3 ± 1.3 A/a	9.88 ± 1.68 A/a	8.40 ± 1.27 A/a	8.82 ± 2.42 A	10.5 ± 3.9 A
	6.7–6.95	(E)-2-hexenal	6728-26-3	854	4.66 ± 0.21 A/a	2.18 ± 0.60 B/a	1.38 ± 0.16 B/a	2.18 ± 0.29 A/b	1.42 ± 0.08 B/a	1.34 ± 0.21 B/a	1.07 ± 0.26 B	1.61 ± 0.42 AB
	8.66–8.92	Heptanal	111-71-7	901	0.40 ± 0.02 C/b	1.40 ± 0.19 B/a	2.44 ± 0.29 A/a	0.78 ± 0.03 B/a	0.81 ± 0.13 B/b	1.09 ± 0.14 AB/b	1.26 ± 0.16 A	1.03 ± 0.13 AB
	11.25–11.45	2-heptenal	18829-55-5	958	0.51 ± 0.05 B/a	0.64 ± 0.02 A/a	0.62 ± 0.03 A/a	0.60 ± 0.07 B/a	0.79 ± 0.11 B/a	0.71 ± 0.13 B/a	1.67 ± 0.09 A	1.65 ± 0.15 A
	13.55	Octenal	124-13-0	1003	0.39 ± 0.05 A/b	0.43 ± 0.10 A/a	0.44 ± 0.07 A/a	0.57 ± 0.04 A/a	0.48 ± 0.12 A/a	0.47 ± 0.10 A/a	0.36 ± 0.06 A	0.43 ± 0.13 A
	16.2	2-octenal	2548-87-0	1060	0.10 ± 0.01 B/b	0.12 ± 0.02 AB/b	0.13 ± 0.01 A/a	0.18 ± 0.02 B/a	0.23 ± 0.07 AB/a	0.27 ± 0.12 AB/a	0.39 ± 0.00 A	0.37 ± 0.03 A
	17.71	(Z)-6-nonenal	2277-19-2	1101	10.7 ± 1.1 B/a	13.8 ± 0.6 A/a	9.74 ± 0.12 B/a	6.74 ± 0.17 A/b	4.93 ± 0.58 B/b	4.44 ± 0.41 BC/b	3.84 ± 0.72 BC	3.25 ± 0.05 C
	17.74	Nonanal	124-19-6	1104	7.04 ± 0.42 A/a	6.47 ± 0.40 AB/a	5.53 ± 0.38 B/a	7.53 ± 0.39 A/a	5.95 ± 1.23 AB/a	5.15 ± 1.01 B/a	3.94 ± 0.44 B	4.26 ± 0.71 B
	18.62–18.68	(E,Z)-2,6-nonadienal	557-48-2	1155	11.8 ± 1.7 A/a	12.9 ± 1.1 A/a	10.9 ± 0.7 A/a	5.71 ± 0.46 B/b	7.25 ± 0.57 B/b	6.69 ± 0.74 B/b	13.9 ± 0.7 A	13.1 ± 0.9 A
	18.7–18.8	(E)-2-nonenal	18829-56-6	1162	9.67 ± 0.40 A/a	11.0 ± 2.1 A/a	10.5 ± 0.4 A/a	5.58 ± 0.59 BC/b	7.19 ± 0.19 ABC/b	4.42 ± 6.99 B/a	14.4 ± 0.4 A	14.0 ± 1.4 AB
	19.37	Decanal	112-31-2	1206	0.58 ± 0.13 A/b	0.52 ± 0.06 A/b	0.62 ± 0.12 A/b	0.99 ± 0.12 A/a	1.01 ± 0.13 A/a	1.29 ± 0.36 A/a	0.66 ± 0.21 A	0.67 ± 0.07 A
	19.46	(E,E)-2,4-nonadienal	5910-87-2	1213	0.09 ± 0.01 B/b	0.13 ± 0.02 A/a	0.14 ± 0.01 A/a	0.13 ± 0.03 C/a	0.16 ± 0.04 C/a	0.20 ± 0.09 BC/a	0.31 ± 0.02 AB	0.40 ± 0.01 A
	S aldehydes				55.4 ± 2.9 A/a	51.8 ± 4.2 AB/a	45.2 ± 0.9 B/a	43.3 ± 2.8 AB/b	39.9 ± 3.94 AB/b	34.5 ± 5.97 B/b	50.6 ± 3.2 A	51.3 ± 6.8 A
**Alcohols**	7.47–7.65	1-hexanol	111-27-3	868	1.57 ± 0.06 B/a	2.00 ± 0.06 B/a	3.70 ± 0.51 A/a	1.44 ± 0.13 A/a	1.37 ± 0.48 A/a	1.41 ± 0.40 A/b	1.63 ± 0.33 A	1.38 ± 0.15 A
	12.07	1-heptanol	111-70-6	970	0.03 ± 0.01 A/b	0.31 ± 0.02 A/a	1.25 ± 0.24 B/a	0.11 ± 0.03 A/a	0.09 ± 0.01 A/b	0.07 ± 0.02 A/b	0.08 ± 0.01 A	0.08 ± 0.05 A
	12.49	1-octen-3-ol	3391-86-4	980	0.04 ± 0.00 C/b	0.07 ± 0.01 B/b	0.12 ± 0.02 A/a	0.14 ± 0.01 B/a	0.15 ± 0.01 B/a	0.16 ± 0.02 B/a	0.35 ± 0.06 A	0.28 ± 0.04 A
	14.86	2-ethyl-1-hexanol	104-76-7	1030	0.14 ± 0.03 A/a	0.07 ± 0.01 B/b	0.13 ± 0.02 A/b	0.20 ± 0.07 B/a	0.54 ± 0.01 B/a	0.47 ± 0.11 B/a	0.18 ± 0.03B	1.11 ± 0.25 A
	16.84	1-octanol	111-87-5	1071	0.24 ± 0.04 B/b	0.30 ± 0.05 B/a	0.47 ± 0.04 A/a	0.42 ± 0.02 A/a	0.39 ± 0.05 A/b	0.33 ± 0.06 A/b	0.45 ± 0.02 A	0.41 ± 0.13 A
	18.6	(Z)-3-nonen-1-ol	10340-23-5	1143	nd	nd	0.48 ± 0.02	0.05 ± 0.00 A	0.06 ± 0.01 A	nd	nd	nd
	18.85	(E,Z)-2,6-nonadien-1-ol	7786-44-9	1169	0.24 ± 0.01 B/a	0.24 ± 0.03 B/a	0.66 ± 0.09 A/a	0.18 ± 0.02 A/b	0.14 ± 0.02 A/b	0.14 ± 0.03 A/b	0.14 ± 0.03 A	0.16 ± 0.05 A
	18.89	(E)-2-nonen-1-ol	31502-14-4	1176	0.37 ± 0.01 B	0.35 ± 0.06 B	0.64 ± 0.09 A	nd	nd	nd	nd	nd
	18.92–18.96	1-nonanol	28473-21-4	1173	2.17 ± 0.09 C/b	4.09 ± 0.20 B/a	8.67 ± 1.01 A/a	5.45 ± 0.31 A/a	3.39 ± 0.74 B/a	3.27 ± 0.53 B/b	3.16 ± 0.55 B	2.57 ± 0.06 B
	S alcohols				4.8 ± 0.2 C/b	7.4 ± 0.3 B/a	16.1 ± 1.8 A/a	7.98 ± 0.47 A/a	6.12 ± 0.39 AB/b	5.81 ± 0.32 B/b	5.99 ± 0.84 B	5.99 ± 0.22 AB
**Furans**	12.98	2-pentyl-furan	3777-69-3	993	0.32 ± 0.03 C/b	0.67 ± 0.04 B/a	0.85 ± 0.02 A/b	0.63 ± 0.05 C/a	1.28 ± 0.45 BC/a	2.63 ± 0.21 A/a	2.39 ± 0.23 A	2.21 ± 0.58 AB
	13.45	cis-2-pentenylfuran	70424-13-4	1002	0.07 ± 0.01 C/b	0.18 ± 0.01 B/a	0.22 ± 0.02 A/b	0.17 ± 0.03 B/a	0.31 ± 0.13 B/a	0.79 ± 0.16 A/a	1.11 ± 0.11 A	1.10 ± 0.17 A
	S furans				0.39 ± 0.04 C/b	0.85 ± 0.05 B/a	1.07 ± 0.04 A/b	0.80 ± 0.07 B/a	1.59 ± 0.58 B/a	3.43 ± 0.03 A/a	3.51 ± 0.26 A	3.32 ± 0.76 A
**Terpenes**	10.08	α-pinene	80-56-8	937	0.09 ± 0.02 B/b	0.12 ± 0.01 B/b	0.20 ± 0.03 A/a	0.25 ± 0.05 A/a	0.32 ± 0.06 A/a	0.25 ± 0.06 A/a	0.15 ± 0.07 A	0.27 ± 0.11 A
	14.6	Limonene	5989-54-8	1030	1.81 ± 2.65 A/a	2.87 ± 4.11 A/a	0.75 ± 0.52 A/a	2.25 ± 3.58 A/a	4.27 ± 0.33 A/a	2.18 ± 2.02 A/a	0.87 ± 0.57 A	2.06 ± 3.11 A
	21.94	α-farnesene	502-61-4	1508	0.13 ± 0.01 B/a	0.14 ± 0.04 B/b	0.24 ± 0.03 A/a	0.11 ± 0.09 B/a	0.31 ± 0.07 AB/a	0.38 ± 0.11 A/a	0.21 ± 0.03 AB	0.32 ± 0.14 AB
	S terpenes				2.03 ± 2.65 A/a	3.12 ± 4.09 A/a	1.19 ± 0.50 A/a	2.61 ± 3.59 A/a	4.89 ± 0.19 A/a	2.81 ± 1.97 A/a	1.23 ± 0.65 A	2.65 ± 3.09 A

^a^ Retention time in min. ^b^ Retention index reported in NIST MS version 2.2. Different letters represent significant differences (*p* < 0.05) at the same conditions (capital letters: effect of storage) or between samples at the same time of storage (noncapital letters: effect of high-pressure processing, HPP).

## Data Availability

The original contributions presented in the study are included in the article/Supplementary Materials, further inquiries can be directed to the corresponding author.

## Supplementary Materials

The following supporting information can be downloaded at: https://www.mdpi.com/article/10.3390/foods13091304/s1, Table S1: Aromatic compounds identified in the samples’ headspace present in the literature; Table S2: Total soluble solids of unprocessed and high-pressure processed fruit salad. The values are expressed as °Brix and presented as mean ± standard deviation; Figure S1: Picture of samples without ascorbic acid taken on the 14th day of storage. The first three, from left to right, are raw samples, while the other three are HPP samples; Figure S2: Picture of samples with ascorbic acid taken on the 14th day of storage. The first three, from left to right, are raw samples, while the other three are HPP samples. References [65,66,67,68,69,70,71,72,73,74,75,76,77] are cited in Supplementary Materials.
