# Supplementary material for: High-Pressure Processing Effects on Microbiological Stability, Physicochemical Properties, and Volatile Profile of a Fruit Salad
Source: Foods. 2024 Apr 24;13(9):1304. doi: 10.3390/foods13091304 (PMC11083073; doi:10.3390/foods13091304)
Supplement: Supplementary file 1 [file foods-13-01304-s001.zip › foods-2782805-supplementary.pdf]

Supplementary materials

Tables

Table S1 – Aromatic compounds identified in the samples' headspace present in the literature.

Compound family	Compound name	Matrix	Reference
Acetate Esters	2-Methyl-1-butanol acetate	Melon	[1]
		Pear	[2]
	Ethyl acetate	Apple	[3,4]
		Melon	[5,6]
		Pear	[7,8]
	Propyl acetate	Melon	[6]
		Pear	[8]
	Butyl acetate	Apple	[9]
		Melon	[1]
		Pear	[7,8]
	Pentyl acetate	Apple	[9]
		Melon	[5]
		Pear	[7]
	Hexyl acetate	Apple	[4,9]
		Melon	[1,6]
		Pear	[8,10]
	2-Hexen-1-ol acetate	Apple	[3]
	Heptyl acetate	Melon	[11]
		Pear	[7,8]
	(Z)- 6-Nonenyl acetate	Melon	[12]
	Nonyl acetate	Melon	[12]
Non-acetate esters	Hexyl 2-methylbutyrate	Apple	[4,13]
Aldehydes	Hexanal	Apple	[13]
		Melon	[5,6,11,14]
		Pear	[8]
	(E)-2-Hexenal	Apple	[13]
		Melon	[5]
		Pear	[8]
	Heptanal	Melon	[5,11,14]
	(E)-2-Heptenal	Melon	[11]
	Octanal	Melon	[11,14]
	(E)-2-Octenal	Melon	[11]
	(Z)-6-Nonenal	Melon	[11]
	Nonanal	Melon	[5,6,11,14]
	(E)-2-Nonenal	Melon	[11]
	(Z)-6-Nonenal	Melon	[11]
	(E,Z)-2,6-Nonadienal	Melon	[11]
	(E,E)-2,4-Nonadienal	Melon	[5,11]
	Decanal	Melon	[5,6,11,14]
Alcohols	1-Hexanol	Apple	[3,9]
		Melon	[11,14]
		Pear	[8,10]
	2-ethyl-1-hexanol	Melon	[6]
	1-Octanol	Melon	[11]
		Pear	[13]
	1-Octen-3-ol	Melon	[15]
	1-Nonanol	Melon	[11]
		Pear	[8]
	(E)-2-Nonen-1-ol	Melon	[15]
		Pear	[7]
	(Z)-3-Nonen-1-ol	Melon	[5,11]
		Pear	[7]
	2,6-Nonadien-1-ol	Melon	[11]
Terpenes	α-pinene	Apple	[16]
	Limonene	Apple	[3]
	α-farnesene	Apple	[4]
		Pear	[7,10]

Table S2 – Total soluble solids of unprocessed and high pressure processed fruit salad. The values are expressed as °brix and presented as mean ± standard deviation.

Sampling day	Raw Samples	ANOVA	HPP samples	ANOVA
**0**	10.80±0.20	A/a	11.07±0.12	A/a
**3**	10.87±0.42	A/a	10.40±0.20	A/a
**7**	10.80±0.35	A/a	10.43±1.01	A/a
**14**	10.20±0.80	A/a	10.93±0.46	A/a
**21**	10.30±0.17	A/a	10.20±0.53	A/a
**28**	10.33±0.64	A/a	10.00±1.06	A/a
**35**	10.73±0.12	A/a	11.27±0.46	A/a

Figures

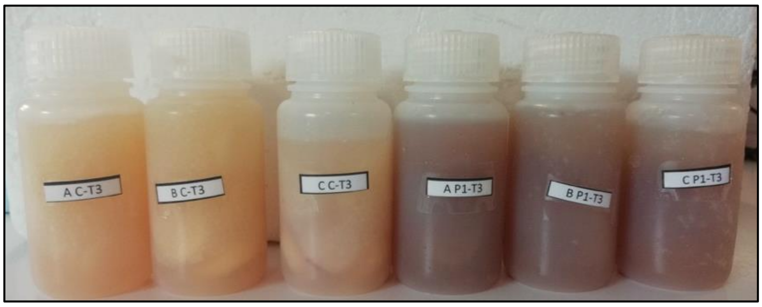

Figure S1 - Picture of samples without ascorbic acid, taken at the 14thday of storage. The first three, from left to right, are raw samples, while the other three are HPP samples.

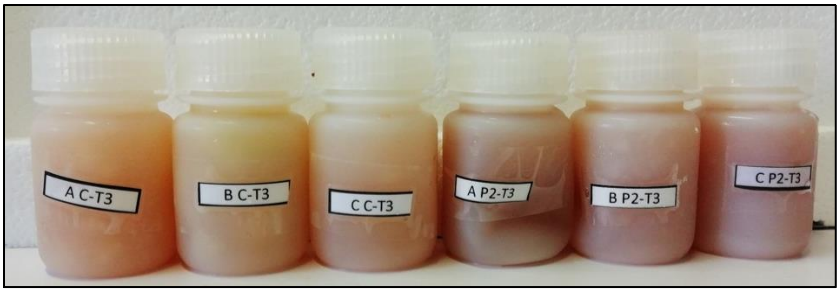

Figure S2 - Picture of samples with ascorbic acid, taken at the 14thday of storage. The first three, from left to right, are raw samples, while the other three are HPP samples.

References

1. Fernández-Trujillo, J.; Dos-Santos, N.; Martínez-Alcaraz, R.; Le Bleis, I. Non-Destructive Assessment of Aroma Volatiles from a Climacteric Near-Isogenic Line of Melon Obtained by Headspace Stir-Bar Sorptive Extraction. *Foods* **2013**, *2*, 401–414, doi:10.3390/foods2030401.
2. Goliáš, J.; Kožíšková, J.; Létal, J. Changes in Volatiles during Cold Storage and Subsequent Shelf-Life of "conference" Pears Treated with 1-MCP. *Acta Horticulturae* **2015**, *1079*, 465–471, doi:10.17660/ActaHortic.2015.1079.61.
3. Giannetti, V.; Boccacci Mariani, M.; Mannino, P.; Marini, F. Volatile Fraction Analysis by HS-SPME/GC-MS and Chemometric Modeling for Traceability of Apples Cultivated in the Northeast Italy. *Food Control* **2017**, *78*, 215–221, doi:10.1016/j.foodcont.2017.02.036.
4. Reis, S.F.A.R.; Rocha, S.M.; Barros, A.S.; Delgadillo, I.; Coimbra, M.A. Establishment of the Volatile Profile of "Bravo de Esmolfe" Apple Variety and Identification of Varietal Markers. *Food Chemistry* **2009**, *113*, 513–521, doi:10.1016/j.foodchem.2008.07.093.
5. Chaparro-Torres, L.A.; Bueso, M.C.; Fernández-Trujillo, J.P. Aroma Volatiles Obtained at Harvest by HS-SPME/GC-MS and INDEX/MS-E-Nose Fingerprint Discriminate Climacteric Behaviour in Melon Fruit. *Journal of the Science of Food and Agriculture* **2016**, *96*, 2352–2365, doi:10.1002/jsfa.7350.
6. Obando-Ulloa, J.M.; Moreno, E.; García-Mas, J.; Nicolai, B.; Lammertyn, J.; Monforte, A.J.; Fernández-Trujillo, J.P. Climacteric or Non-Climacteric Behavior in Melon Fruit. 1. Aroma Volatiles. *Postharvest Biology and Technology* **2008**, *49*, 27–37, doi:10.1016/j.postharvbio.2007.11.004.
7. Chen, Y.; Yin, H.; Wu, X.; Shi, X.; Qi, K.; Zhang, S. Comparative Analysis of the Volatile Organic Compounds in Mature Fruits of 12 Occidental Pear (*Pyrus Communis* L.) Cultivars. *Scientia Horticulturae* **2018**, *240*, 239–248, doi:10.1016/j.scienta.2018.06.014.
8. Taiti, C.; Marone, E.; Lanza, M.; Azzarello, E.; Masi, E.; Pandolfi, C.; Giordani, E.; Mancuso, S. Nashi or Williams Pear Fruits? Use of Volatile Organic Compounds, Physicochemical Parameters, and Sensory Evaluation to Understand the Consumer's Preference. *European Food Research and Technology* **2017**, *243*, 1917–1931, doi:10.1007/s00217-017-2898-y.
9. Lee, J.; Jang, H.W.; Jeong, M.C.; Yoo, S.R.; Ha, J. Analysis of Volatile Compounds as Quality Indicators for Fuji Apples after Cold Storage. *Journal of Food Biochemistry* **2017**, *41*, 1–11, doi:10.1111/jfbc.12410.
10. Yi, X.K.; Liu, G.F.; Rana, M.M.; Zhu, L.W.; Jiang, S.L.; Huang, Y.F.; Lu, W.M.; Wei, S. Volatile Profiling of Two Pear Genotypes with Different Potential for White Pear Aroma Improvement. *Scientia Horticulturae* **2016**, *209*, 221–228, doi:10.1016/j.scienta.2016.06.034.
11. Fredes, A.; Sales, C.; Barreda, M.; Valcárcel, M.; Roselló, S.; Beltrán, J. Quantification of Prominent Volatile Compounds Responsible for Muskmelon and Watermelon Aroma by Purge and Trap Extraction Followed by Gas Chromatography-Mass Spectrometry Determination. *Food Chemistry* **2016**, *190*, 689–700, doi:10.1016/j.foodchem.2015.06.011.
12. Engel, K.; Heidlas, J.; Tressl, R. The Flavour of Tropical Fruits (Banana, Melon, Pineapple). In *Food Flavours. Part C. The Flavour of Fruits*; Morton, I.D., Macleod, A.J., Eds.; Elsevier B.V.: Amsterdam, 1990; pp. 201–206 ISBN 0-444-87362-7.
13. Paillard, N.M.M. The Flavour of Apples, Pears and Quinces. In *Food Flavours. Part C. The Flavour of Fruits*; Morton, I.D., Macleod, A.J., Eds.; Elsevier B.V.: Amsterdam, 1990; pp. 1–34 ISBN 0-444-87362-7.
14. Fernández-Trujillo, J.; Zarid, M.; Bueso, M.C. Methodology to Remove Strong Outliers of Non-Climacteric Melon Fruit Aroma at Harvest Obtained by HS-SPME GC-MS Analysis. *Separations* **2018**, *5*, doi:10.3390/separations5020030.
15. Oh, S.H.; Lim, B.S.; Hong, S.J.; Lee, S.K. Aroma Volatile Changes of Netted Muskmelon (*Cucumis Melo* L .) Fruit during Developmental Stages. **2011**, *52*, 590–595, doi:10.1007/s13580-011-0090-z.
16. Burdock, G.A. *Fenaroli's Handbook of Flavor Ingredients*; 4th ed.; CRC Press LLC: Boca Raton, Florida, 2002; ISBN 0-8493-0946-8.
